# CISH constrains the tuft–ILC2 circuit to set epithelial and immune tone

**DOI:** 10.1038/s41385-021-00430-6

**Published:** 2021-07-21

**Authors:** Maya E. Kotas, Nicholas M. Mroz, Satoshi Koga, Hong-Erh Liang, Andrew W. Schroeder, Roberto R. Ricardo-Gonzalez, Christoph Schneider, Richard M. Locksley

**Affiliations:** 1 Division of Pulmonary, Critical Care, Allergy & Sleep Medicine, University of California, San Francisco, CA USA; 2 Department of Laboratory Medicine, University of California, San Francisco, CA USA; 3 Department of Medicine, University of California, San Francisco, CA USA; 4 Genomics CoLab, University of California, San Francisco, CA USA; 5 Department of Dermatology, University of California, San Francisco, CA USA; 6 Institute of Physiology, University of Zürich, Zürich, Switzerland; 7 Howard Hughes Medical Institute, University of California, San Francisco, CA USA

## Abstract

Innate lymphoid cells (ILCs) are tissue-resident effectors poised to activate rapidly in response to local signals such as cytokines. To preserve homeostasis, ILCs must employ multiple pathways, including tonic suppressive mechanisms, to regulate their primed state and prevent inappropriate activation and immunopathology. Such mechanisms remain incompletely characterized. Here we show that cytokine-inducible SH2-containing protein (CISH), a suppressor of cytokine signaling (SOCS) family member, is highly and constitutively expressed in type 2 innate lymphoid cells (ILC2s). Mice that lack CISH either globally or conditionally in ILC2s show increased ILC2 expansion and activation, in association with reduced expression of genes inhibiting cell-cycle progression. Augmented proliferation and activation of CISH-deficient ILC2s increases basal and inflammation-induced numbers of intestinal tuft cells and accelerates clearance of the model helminth, *Nippostrongylus brasiliensis*, but compromises innate control of *Salmonella typhimurium*. Thus, CISH constrains ILC2 activity both tonically and after perturbation, and contributes to the regulation of immunity in mucosal tissue.

## Introduction

Innate lymphoid cells (ILCs) are poised effectors that are seeded into peripheral tissues during fetal development, where they can subsequently activate rapidly in response to local perturbations. Three major subsets of ILCs have been identified, classified according to their cytokine outputs and their similarity to the major subsets of helper T cells: type 1 innate lymphoid cells (ILC1s) which share features and function with type 1 helper T cells (Th1); type 3 innate lymphoid cells (ILC3s) which resemble Th17 cells; and type 2 innate lymphoid cells (ILC2s), which share similarities with Th2 cells.^1^ ILC2s are activated by diverse signals, including eicosanoids, neuropeptides, and hormones, as well as cytokines like interleukin (IL)-25, IL-33, IL-18, and thymic stromal lymphopoietin (TSLP), to rapidly secrete type 2 cytokines like IL-13, IL-9, GM-CSF, and IL-5.^1,2^

The differentiation and positioning of ILCs in peripheral tissues enables them to respond quickly to local perturbations by recruiting other immune cells and inducing coordinated responses among stromal and epithelial cells. Lacking the antigen-specific receptors used by adaptive lymphocytes, ILCs require alternative mechanisms to ensure activation is limited to appropriate contexts. Natural killer (NK) cells, ILC1s, and NK cell receptor (NCR) + ILC3s utilize a well-characterized array of activating and inhibitory surface receptors that provide antigen-independent checkpoints to activation.^3,4^ Similarly, ILC2s are inhibited by a variety of exogenous cues, such as ligation of KLRG1,^1^ cytokines such as IL-12, IL-27, or type I or II interferons,^5,6^ and neurotransmitters such as calcitonin gene-related peptide (CGRP)^7^ and adrenergics.^8^ However, apart from A20, which negatively regulates IL-25 signaling,^9^ endogenous mechanisms that regulate ILC2 activity are less well characterized.

The SOCS proteins include 8 members (SOCS1-7 and CISH) that are upregulated in response to cytokines or other activating signals to negatively regulate the magnitude and duration of such signals. SOCS proteins inhibit cytokine signaling through various mechanisms, including inactivation of Janus kinases (JAKs) downstream of cytokine receptors, interfering with binding of signal transducers and activators of transcription (STATs) to cytokine receptors, and ubiquitination and proteosomal degradation of signaling intermediates.^10^ CISH, also called CIS, was first identified as an early response gene induced by growth factors such as erythropoietin, thrombopoietin, IL-2, and IL-3 that signal through STAT5.^11^ CISH blunts signaling downstream of these receptors in part by blocking STAT5 binding to the relevant cytokine receptors, and by promoting receptor complex ubiquitination and degradation.^11–13^ Subsequent investigations have also identified non-STAT5-dependent stimuli that induce CISH expression in diverse cell types,^10^ including following T cell receptor (TCR) ligation where CISH is thought to dampen TCR signaling through interactions with phospholipase C (PLC)-γ1.^14^

Consistent with the general inhibitory functions of SOCS proteins, studies of mice that are completely or selectively deficient in CISH have shown increased activation of targeted cell types. Conditional knockout of CISH led to increased interferon-γ production and augmented anti-tumor responses in CD8+ T cells^14^ or NK cells.^15^ In the case of NK cells, this related to enhanced metabolic fitness that favored survival and proliferation under conditions of limiting cytokines like IL-2 and IL-15.^16^ In addition to generalized inhibition, some reports suggest that CISH imparts differential control across activation programs in the same cells. For instance, selective knockout of CISH in dendritic cells augmented their proliferation but reduced their ability to activate cytotoxic T lymphocytes.^17^ Curiously, mice with global CISH deficiency developed spontaneous allergic pulmonary disease as they age, and selective knockout of CISH in CD4+ T cells led to T cell hyperactivation with a bias towards Th2/Th9 differentiation.^18^ Thus, CISH appears to regulate many outputs, but may be particularly important in vivo in constraining type 2 polarization. With this background, we sought to characterize the role of CISH in ILC2s.

## Results

### *Cish* is highly and constitutively expressed in tissue-resident ILC2s

**Fig. 1:**
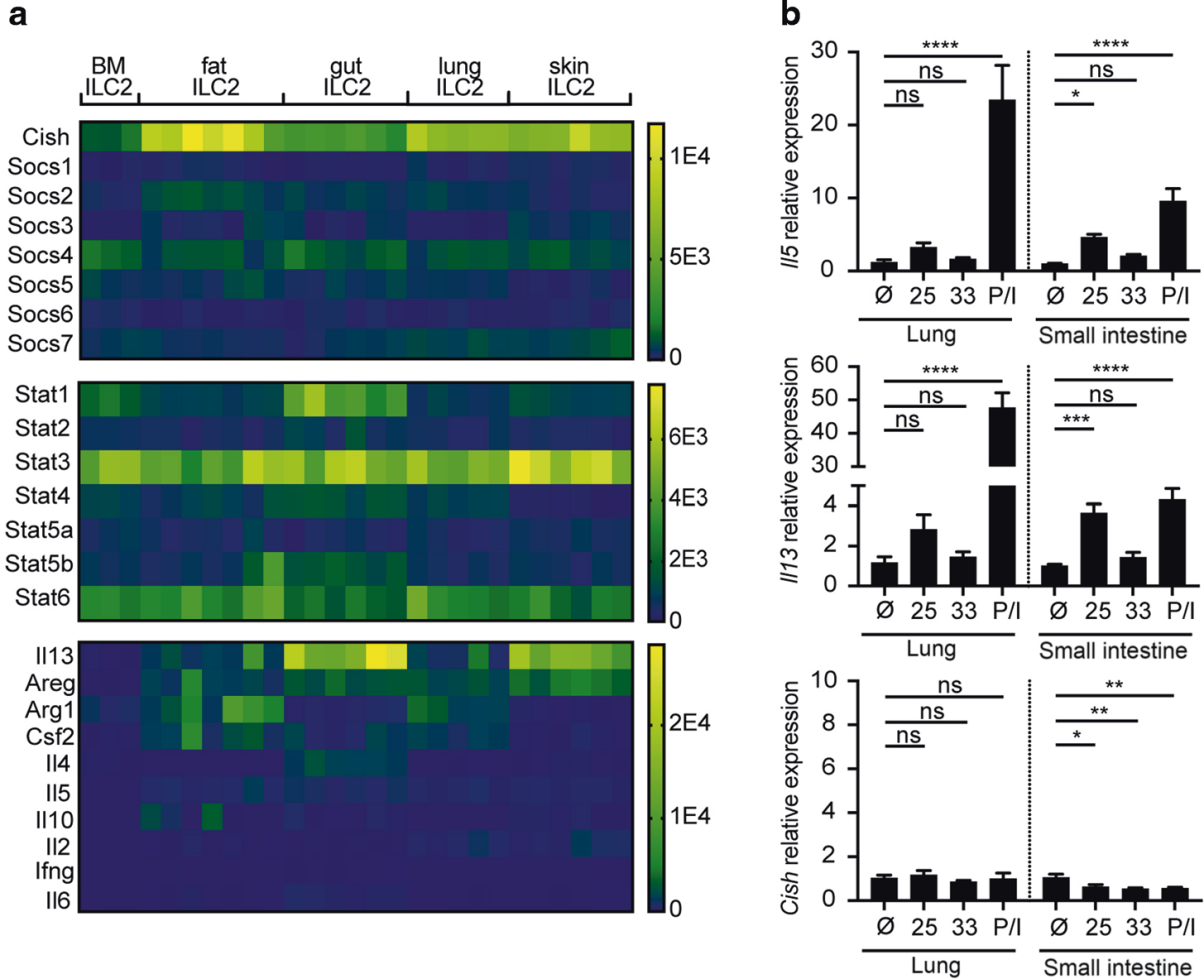
CISH is highly and constitutively expressed in tissue resident ILC2s. **a** RNA sequencing of purified ILC2s from indicated tissues. Legend colors indicate counts per million reads. **b** Induction of *Il5* and *Il13* but not *Cish* in cultured ILC2s from lung or SI treated with 10 ng/mL of IL-25 or IL-33, or PMA/ionomycin.*p < 0.05, **p < 0.01, ***p < 0.001, ****p < 0.0001 for one-way ANOVA with Dunnett testing for multiple comparisons. *n* = 3 wells/stimulation, each derived from a single pool of ILC2s isolated from 6 mice.

Analysis of datasets at ImmGen^19^ revealed that *Cish* is highly expressed in ILC2s as compared to other tissue-resident lymphocytes (Supplementary Fig. 1a). To validate and further explore this observation, we mined bulk RNA sequencing of sorted ILC2s and other lymphocytes previously performed in our lab.^20^ *Cish* was the most highly expressed SOCS family member in ILC2s in all tissues examined (Fig. 1a). *Cish* was also highly expressed in ILC2s as compared to CD4+ T cells or Tregs (Supplementary Fig. 1b, c), independently corroborating the ImmGen data. Expression of *Cish* was higher in ILC2s from peripheral tissues than in ILC2s from bone marrow and accompanied the increased expression of canonical ILC2 effector genes (Fig. 1a). Because CISH and other SOCS family proteins interfere with JAK-STAT signaling in multiple cell types, we also examined STAT family transcripts in ILC2s. *Stat1* transcripts correlated inversely with *Cish* transcripts across tissue ILC2s (Fig. 1a).

Because *Cish* was initially discovered as an early inducible transcript in stimulated peripheral blood mononuclear cells, we considered whether CISH might be induced in ILC2s during stimulation. ILC2s can be activated by multiple cytokines, including TSLP, IL-25, and IL-33, which have been shown to coordinately regulate the basal output of ILC2s in vivo.^20^ However, *Cish* expression in tissue ILC2s isolated from mice lacking signaling from these three cytokines (“TKO”) was not consistently decreased compared to ILC2s from WT mice (Supplementary Fig. 1d). Consistent with this finding, when we stimulated ILC2s from lung or small intestine with IL-25, IL-33, or PMA/ionomycin ex vivo, transcripts for prototypical ILC2 effector cytokines such as IL-5 and IL-13 were readily induced by activation, whereas *Cish* mRNA was not (Fig. 1b). Thus, CISH is highly and constitutively expressed in tissue ILC2s and not further induced following activation by tissue cytokines under these conditions.

### CISH deficiency in ILC2s augments ILC2 responses and accelerates helminth clearance

**Fig. 2:**
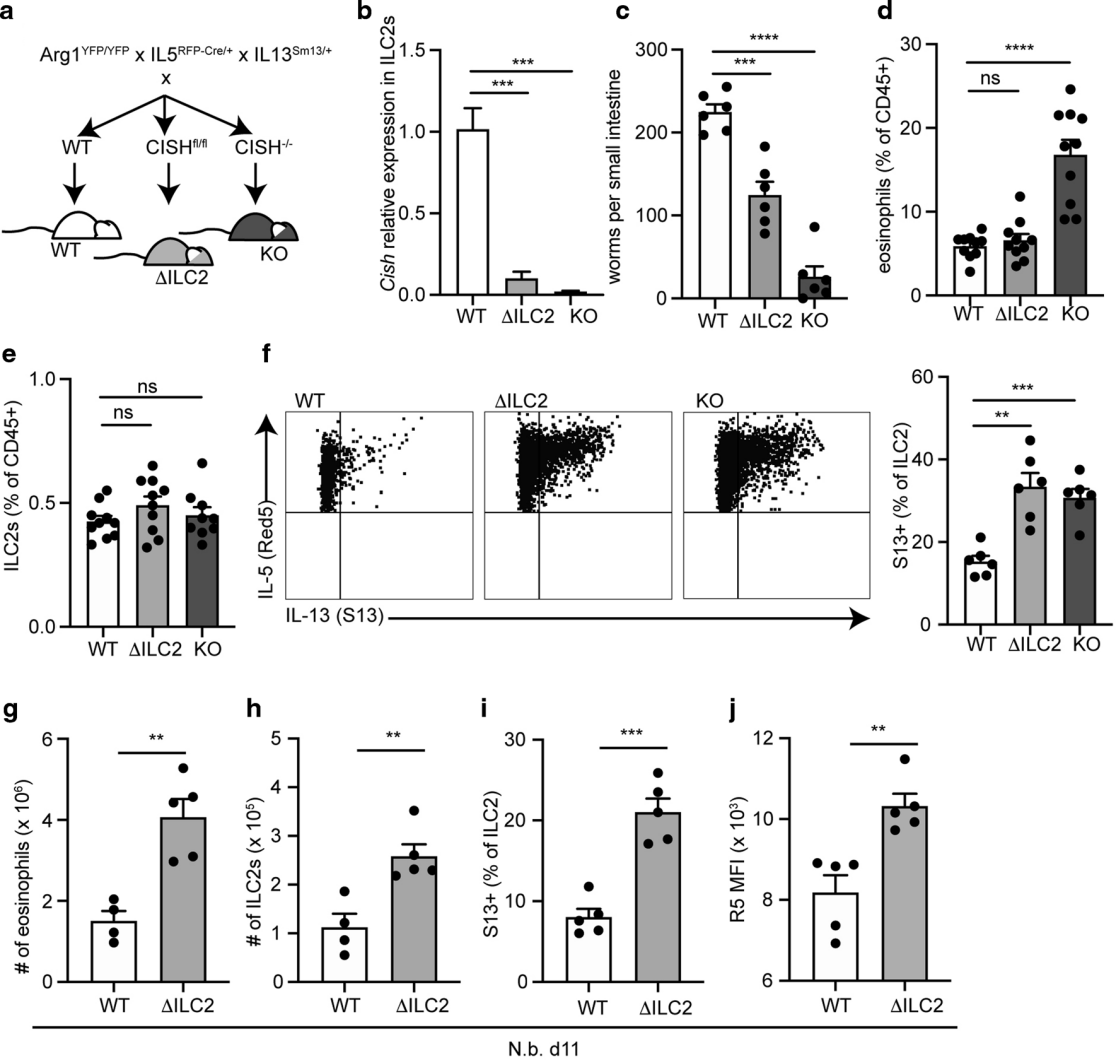
Knockdown of CISH in ILC2s leads to augmented ILC2 responses and accelerated helminth clearance. **a** Breeding strategy using IL5^RFP-Cre^ (R5) to report on IL-5 expression and conditionally knockout CISH on ILC2s. **b** Efficient R5-mediated deletion of *Cish* mRNA on sorted lung ILC2s. ***p < 0.001 for one-way ANOVA with Dunnett testing for multiple comparisons. *n* = 3 mice/group. **c** Number of small intestinal *N.b*. worms counted on day 5 of infection with N.b. ***p < 0.001, ****p < 0.0001 for one-way ANOVA with Dunnett testing for multiple comparisons. *n* = 6 mice/group. Representative of at least 3 independent experiments. **d** Eosinophils from lungs of *N.b*.-infected mice of indicated genotypes on day 5. Eosinophils were gated as CD45+, SigF+, CD11b+, live cells. ***p < 0.001 for Brown–Forsythe and Welch ANOVA with Dunnett correction for multiple comparison. “ns” non-significant. *n* = 8–10 mice/group. Represents combined data from 2 of 3 similar experiments. **e** ILC2s from lungs of *N.b*.-infected mice on day 5 as in **d**. *n* = 8–10 mice/group; represents combined data from 2 of 3 similar experiments. **f** Representative flow cytometry plots (left panels) and corresponding quantification (bar graph, right panel) of S13 expression on ILC2s from mice in **c**–**e**. ILC2s were gated as lin–, Thy1.2+, Arg1^YFP^+, R5+. ***p < 0.001, **p < 0.01 for Brown–Forsythe and Welch ANOVA with Dunnett correction for multiple comparison. “ns” non-significant. *n* = 6 mice/group. Representative of at least 3 similar experiments. **g** Number of eosinophils from lungs of *N.b.-*infected mice on day 11. **h** Number of ILC2s from lungs of *N.b*.-infected mice on day 11. **i** S13 expression on ILC2s from lungs of *N.b*.-infected mice on day 11. **j** R5 MFI from d11 *N.b*.-infected mice. For (**g**–**j**), **p < 0.01, ***p < 0.001 by 2-tailed *t*-test. *n* = 5 mice/group.

To examine the role for CISH in modulating ILC2 responses, we examined cells from tissues with global CISH deficiency, or with CISH-deficiency in IL-5+ cells, which largely consist of ILC2s in uninfected mice (Fig. 2a). Deletion of CISH in ILC2s using IL-5-Cre was efficient (Fig. 2b, Supplementary Fig. 2a), and did not result in compensatory upregulation of other SOCS family members (Supplementary Fig. 2b). We challenged ILC2 conditional CISH knockout mice (“ΔILC2”) or global CISH knockout mice (“KO”) with the parasitic helminth *Nippostrongylus brasiliensis* (*N.b*.) to activate ILC2s.^21^ As assessed 5 days after infection, KO mice had almost completely cleared intestinal organisms, while wildtype (WT) mice had ~200 remaining worms (Fig. 2c). ΔILC2 mice exhibited accelerated worm clearance that was intermediate between WT and KO mice at this timepoint (Fig. 2c). Eosinophils, which are recruited and activated by ILC2s,^22–24^ were significantly increased in lungs from infected KO mice as compared to WT mice, although this did not occur in ΔILC2 mice at this time point (Fig. 2d).

The frequency of lung ILC2s in infected mice was not yet increased in CISH-deficient mice by day 5 (Fig. 2e), and similar to that in infected WT mice.^21,25^ However, ILC2s from both ΔILC2 and KO mice had increased production of IL-13 (Fig. 2f) and ΔILC2 and KO mice had increased plasma IL-5 at day 5 as compared to WT mice (Supplementary Fig. 2c). By day 11 of infection, a timepoint that approaches peak ILC2 activation in the lung of WT mice,^26^ infected ΔILC2 mice had increased lung eosinophils, increased ILC2 numbers, and increased IL-5 and IL-13 expression by lung ILC2s as compared to WT mice, consistent with a role for CISH in restraining ILC2 activity in response to tissue perturbation (Fig. 2g–j).

Because conditional knockout of CISH in ILC2s only partially recapitulated the accelerated *N.b*. larval clearance observed in global KOs (Fig. 2c), and because T cells are also known to be important for clearance of *N.b*.,^21^ we examined a role for T cells as targets of regulation by CISH. Mice with CD4-Cre-driven conditional knockout of CISH in T cells (“ΔCD4”) also had an intermediate reduction in the level of intestinal worms at day 5 (Supplementary Fig. 2d), resembling that seen in the infected ΔILC2 mice. Like deletion of CISH in ILC2s, deletion of CISH in T cells did not lead to the increase in early pulmonary eosinophils observed in infected CISH KO mice (Supplementary Fig. 2e). In contrast to CISH-deficient ILC2s, neither ILC2s nor CD4+ T cells from infected ΔCD4 mice exhibited increased IL-13 expression (Supplementary Fig. 2f).

### Deficiency of CISH in ILC2s leads to an increase in intestinal tuft cells

**Fig. 3:**
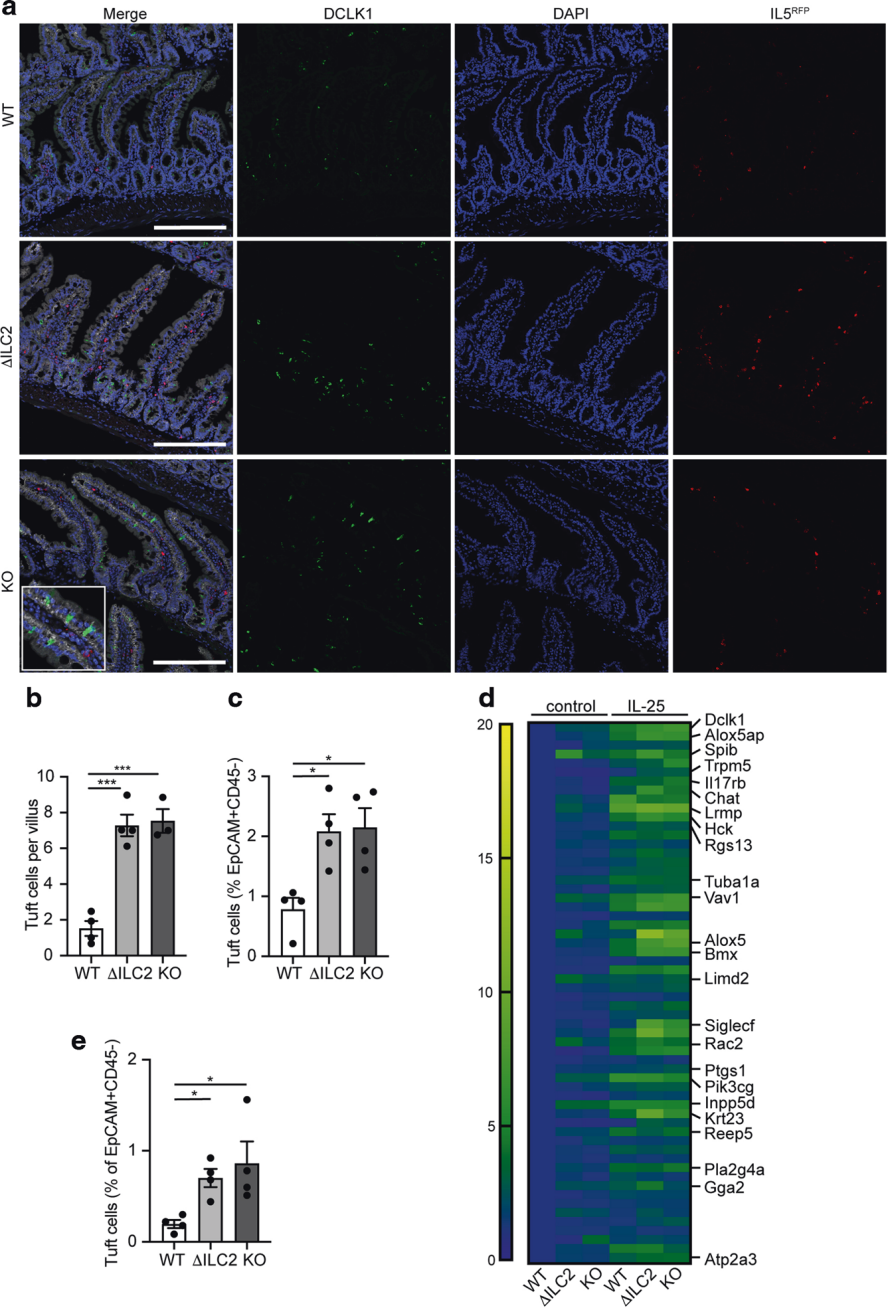
Loss of CISH in ILC2s leads to an increase in intestinal tuft cells. **a** Representative images of small intestine from indicated strains of mice sacrificed on day 4 following 3 daily doses of i.p. IL-25 treatment. EpCAM (white), DCLK1 (green), IL5^RFP^ + ILC2s (red), DAPI (blue). Scale bars represent 200 μm. **b** Quantification of tuft cell numbers per villus as shown by immunofluorescence in **a**. ***p < 0.001 for one-way ANOVA with Dunnett testing for multiple comparisons. *n* = 3-4 mice/group. **c** Flow cytometric quantification of tuft cells (DCLK1+, EpCAM+, CD44–, CD45–, live cells) in epithelial fraction of jejunum of IL-25-treated mice. *p < 0.05 for one-way ANOVA with Dunnett testing for multiple comparisons. *n* = 4 mice/group. **d** Heatmap of tuft cell consensus signature in untreated or IL-25-treated mice of indicated genotypes. Each block represents row-normalized mean expression of 3 mice/group. **e** Flow cytometric quantification of tuft cells (DCLK1+, EpCAM+, CD44–, CD45–, live cells) in epithelial fraction of jejunum of untreated mice by flow cytometry. *p < 0.05 for one-way ANOVA with Dunnett testing for multiple comparisons. *n* = 4 mice/group. Representative of 3 similar experiments.

Intestinal expulsion of *N.b*. is accomplished through coordinated cross-talk between epithelial tuft cells and ILC2s, which together form an amplifying immune circuit.^27–29^ In this circuit, ILC2-derived IL-13 drives the intestinal epithelium towards secretory lineages, increasing the number of tuft and goblet cells, and resulting in the “weep and sweep” response that accompanies parasite clearance. We hypothesized that CISH deficiency in ILC2s may contribute to accelerated helminth clearance by augmenting tuft cell activity or numbers in the small intestine. Quantifying tuft cells from the proximal intestine of *N.b*.-infected ΔILC2 mice revealed a trend towards increased tuft cell numbers at the early phase (day 3) of the infection that was lost at the peak of tuft cell accumulation (day 11) (Supplementary Fig. 3a). Since small intestinal tuft cell numbers reflect the activation of ILC2s by IL-25 from tuft cells themselves,^27,30^ we directly assessed the effects of CISH deficiency after treatment with exogenous IL-25, as previously described.^26,31^ Indeed, ΔILC2 or global KO mice treated with 3 daily doses of IL-25 had increased numbers of jejunal tuft cells compared to WT mice (Fig. 3a–c). Corroborating this finding, we observed that genes associated with a consensus tuft cell signature^32^ were increased in whole jejunal tissue of IL-25-treated ΔILC2 or global KO mice when compared to WT mice (Fig. 3d, Supplementary Table 3). Additionally, we observed that tuft cell-associated genes were already elevated in untreated ΔILC2 or KO mice compared to WT mice (Fig. 3d), and were further augmented after IL-25 treatment. Indeed, tuft cells were increased 3-fold in untreated ΔILC2 or KO mice compared to WT mice (Fig. 3e). A similar increase in goblet cells and associated transcripts was also evident in ΔILC2. conditional or KO mice (Supplementary Fig. 3b, c, Supplementary Table 3). Collectively, these data suggest that small intestinal epithelial secretory lineages influenced by ILC2-derived IL-13 are enhanced in ILC2- or globally CISH-deficient mice.

### Deficiency of CISH in ILC2s leads to increased ILC2 activity during homeostatic conditions

Having found direct evidence of increased ILC2 activation following perturbation and indirect evidence of increased ILC2 activation at rest (as indicated by the augmented numbers of tuft cells) in the absence of CISH, we examined ILC2s under basal conditions for evidence of increased activity. We observed no differences in the frequency of ILC2s in the unperturbed state in any of the tissues examined (Supplementary Fig. 4a). Additionally, neither conditional nor global CISH knockout mice had altered percentages of IL-13+ resident ILC2s (Supplementary Fig. 4b), suggesting a similar degree of activation in CISH-deficient and sufficient ILC2s across tissues in situ. Corroborating this, sorted lung ILC2s from conditional CISH knockout mice had unaltered mRNA expression of prototypical ILC2 effector genes, such as *Il13*, *Il5*, *Csf2*, and *Areg* (Supplementary Fig. 4c). Despite these similarities in numbers and cytokine outputs, however, we found that CISH-deficient ILC2s were more proliferative (Supplementary Fig. 4d). The numbers of other tissue immune cells that may be influenced by ILC2s, including alveolar macrophages, eosinophils, and CD4+ or CD8+ T cells, were unaltered in conditional or global CISH knockout mice (Supplementary Fig. 4e).

**Fig. 4:**
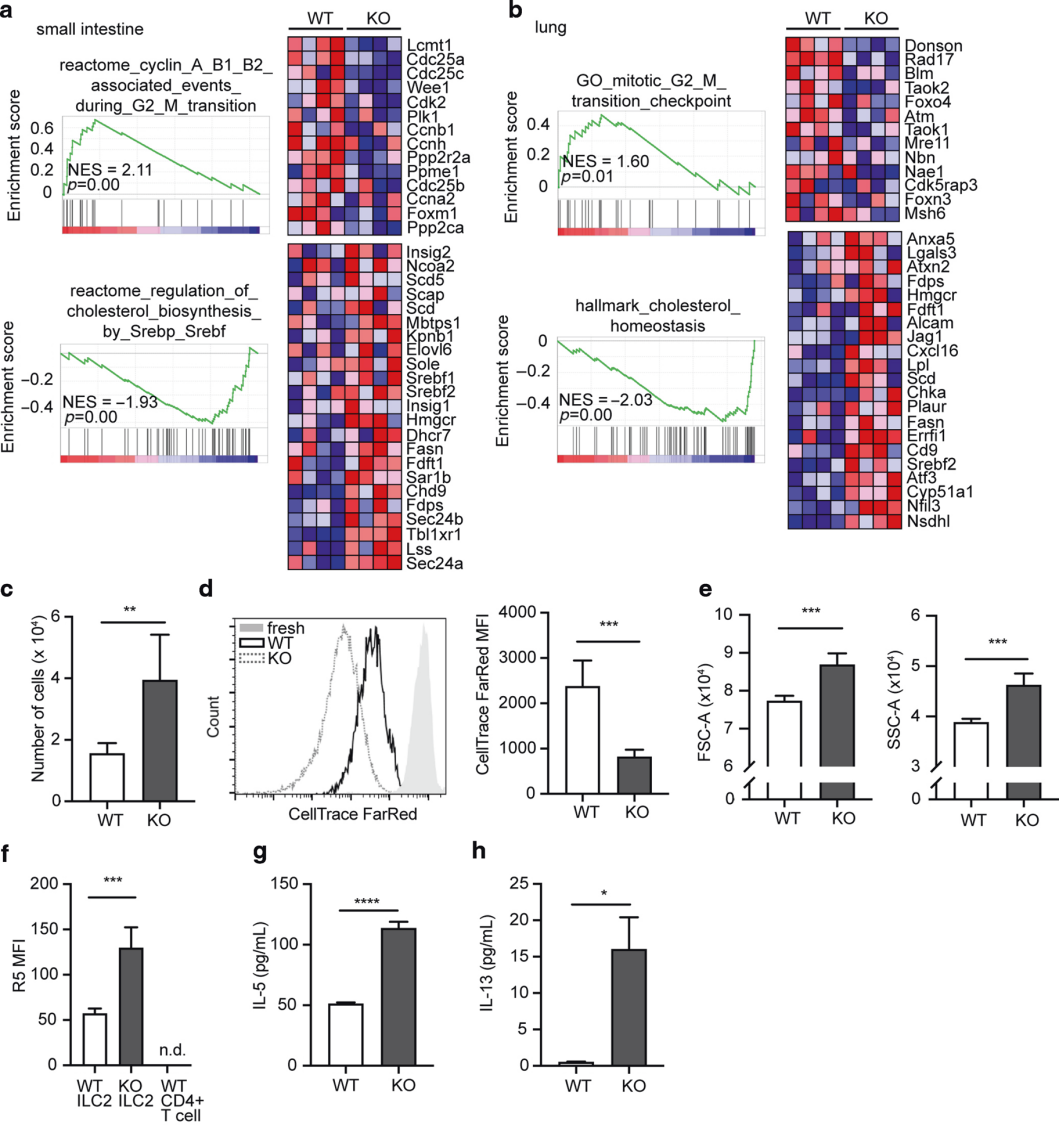
CISH constrains proliferation and cytokine production in ILC2s. **a** Gene set enrichment analysis of small intestinal ILC2s from WT or CISH KO mice. **b** Gene set enrichment analysis of lung ILC2s from WT or CISH KO mice. For **a** and **b**, each column represents sequenced cells from an individual mouse. Color blocks indicate row-normalized expression. *n* = 4 mice/group. **c** Number of live ILC2s after 1 week of culture in 10 ng/mL IL-2 and IL-7. **p < 0.01 by unpaired *t*-test. for **c**–**f**, *n* = 5 wells/group with each well derived from a single mouse. **d** Histogram (left) and graphical quantitation (right) of CellTrace Far Red dye dilution in cultured ILC2s. “fresh” indicates cells immediately after staining, without subsequent culture. ***p < 0.001 by unpaired *t*-test. **e** Forward scatter (FSC) and side scatter (SSC) of cultured ILC2s after 1 week of culture. ***p < 0.001 by unpaired *t*-test. **f** R5 MFI in cultured ILC2s. ***p < 0.001 by unpaired *t-*test. **g** IL-5 from culture supernatant of cells cultured for 4d in IL-2 and IL-7. ****p < 0.0001 by unpaired *t*-test. *n* = 4 wells/group, derived from a split pooled collection of ILC2s from 8 mice/genotype. **h** IL-13 from culture supernatant of cells cultured for 4d. *p < 0.05 by unpaired *t*-test. *n* = 4 wells/group.

To address the difference between CISH-sufficient and -deficient ILC2s more granularly, we performed RNA sequencing of sorted WT or CISH-deficient ILC2s from small intestine and lung. Pathway analysis revealed shared perturbations among ILC2s between tissues, including an enrichment of cell-cycle control genes (*e.g., Cdc25a, Cdc25c*) in WT compared to KO cells (Fig. 4a, b, Supplementary Table 4). Upregulated pathways in KO ILC2s included those associated with cholesterol biosynthesis, such as *Hmgcr*, which encodes the rate-limiting enzyme 3-hydroxy-3-methylglutaryl-CoA reductase, and the transcriptional regulators *Srebf1* and *Srebf2* (Fig. 4a, b). These changes are in line with the essential need for cholesterol production for cell-cycle progression, including in lymphocytes.^33–37^ To test the proliferative capacity of CISH-deficient ILC2s, we cultured purified ILC2s in vitro for 1 week with IL-2 and IL-7. Under these conditions, KO ILC2s proliferated more (Fig. 4c, d), grew larger and more granular (Fig. 4e), and produced more IL-5 (Fig. 4f, g) and IL-13 (Fig. 4h). Cumulatively, these data suggest that CISH intrinsically restricts proliferation and activation of ILC2s in vitro and in vivo.

### Loss of CISH in ILC2s leads to dysregulated immune responses to diverse pathogens

**Fig. 5:**
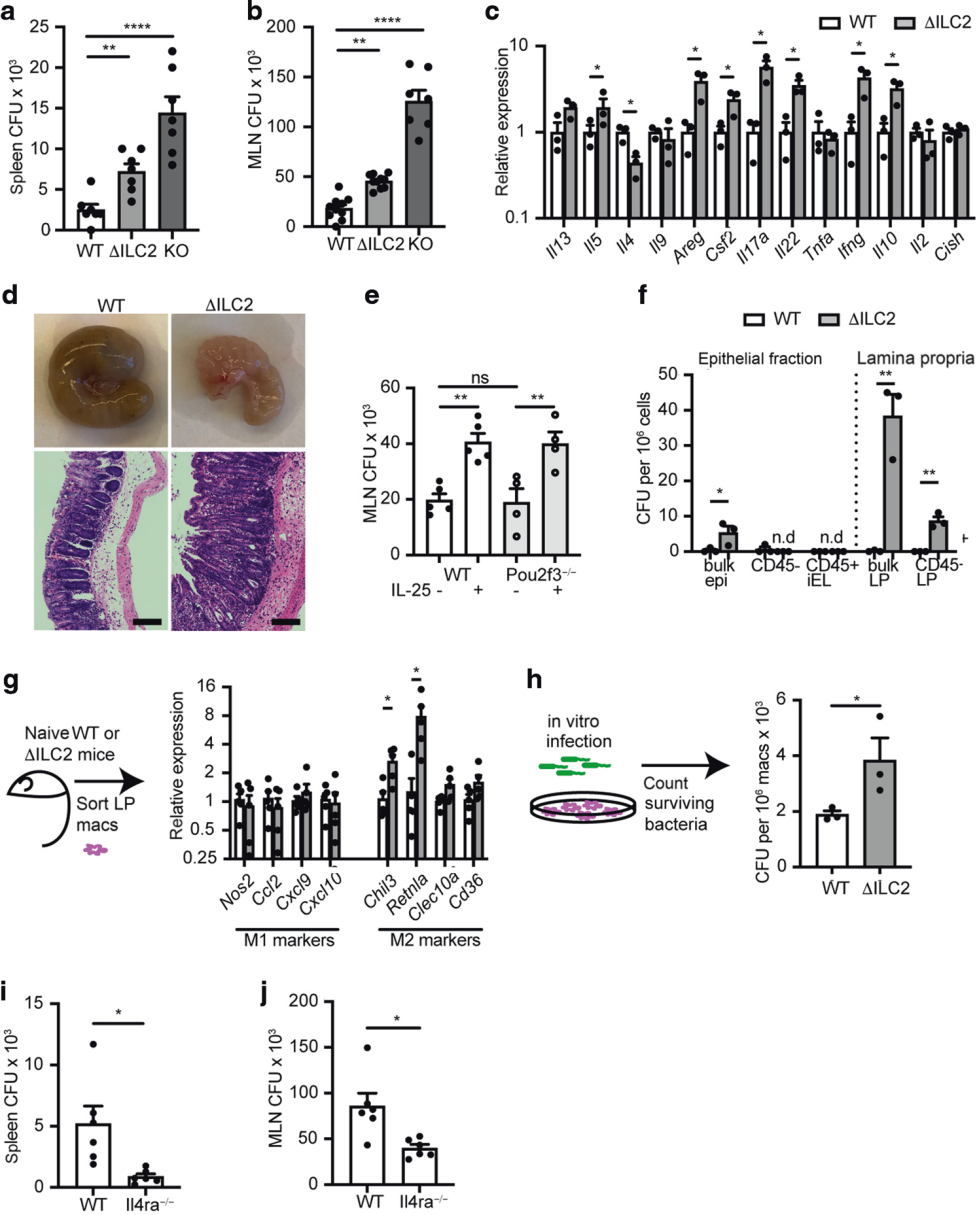
Loss of CISH in ILC2s impairs control of *Salmonella* infection. **a** Colony forming units (CFU) of *S. typhimurium* in spleens of indicated mice on day 3 of infection. **b** CFU from mesenteric lymph nodes (MLN) of indicated mice at day 3. for **a** and **b**, *p < 0.05, **p < 0.01, ***p < 0.001, ****p < 0.0001 for one-way ANOVA with Dunnett testing for multiple comparisons. *n* = 7–10 mice/group, pooled from 2 of 3 independent experiments. **c** Cytokine transcripts from dissociated MLNs of indicated genotype collected on day 3 of infection and cultured overnight. *p < 0.05 by *t*-test. unannotated comparisons not significant. *n* = 3 mice/group. **d** Representative gross appearance and H&E-stained histologic appearance of ceca from indicated strains. Scale bars represent 100 μm. **e** CFU from MLN of WT (closed circles, white bars) or Pou2f3^−/−^ (open circles, gray bars) mice treated with PBS or IL-25 to activate ILC2s. **p < 0.01 by *t*-test. *n* = 4-5 mice/group. **f** CFU from epithelial-enriched fraction (bulk epi) or sorted CD45– (predominantly epithelial cells) or CD45+ (intraepithelial leukocytes, “iEL”) populations from the epithelial-enriched fraction (left of dotted line); or from the bulk lamina propria-enriched fraction or FACS-sorted CD45+ lamina propria (LP) cells (right of dotted line) of WT or ΔILC2 mice. n.d. not detected. *p < 0.05, **p < 0.01 by *t*-test. *n* = 3 mice/group. **g** mRNA expression of M1 and M2 markers in FACS-sorted lamina propria macrophages from uninfected (naïve) WT (clear bars) or ΔILC2 (gray bars) mice. *p < 0.05. unannotated comparisons not significant. *n* = 4 mice/group. **h** FACS-sorted lamina propria macrophages from uninfected (naïve) WT (clear bars) or ΔILC2 (gray bars) were infected in vitro with *S. typhimurium*, cleared of extracellular bacteria, and lysed to quantify surviving/intracellular bacterial CFUs. *p < 0.05 by *t*-test. *n* = 3 mice/group. **i** CFU of *S. typhimurium* in spleens of indicated genotypes of mice on day 3 of infection. j CFU from MLN of indicated mice at day 3. for **i** and **j**, *p < 0.05 by *t*-test. *n* = 6 mice/group.

To optimize overall fitness, the canonical effector outputs of the immune system exist in careful balance.^2^ Persistent perturbation of one output—in this case, a propensity towards type 2 activation—is likely to impact the function of the others.^38^ To examine the effects of augmented ILC2 activity induced by CISH deficiency, we challenged WT, ΔILC2 conditional, or KO mice with oral *Salmonella enterica* serovar *typhimurium (S. typhimurium)* infection, a model of lethal foodborne illness that induces secretion if IFN-γ, IL-22, and IL-17, among other cytokines, to control early infection.^39^ Three days after infection, KO mice had a significantly increased bacterial burden in spleen and mesenteric lymph nodes (MLN) compared to WT mice, whereas ΔILC2 mice exhibited bacterial burdens that were intermediate between WT and KO mice (Fig. 5a, b). No significant differences were observed in major immune cell types—such as monocytes, CD8+ T cells, NK cells, CD4+ T cells, granulocytes, or ILC2s—within the lamina propria (Supplementary Fig. 5a), MLN, or spleen (not shown). However, when we cultured bulk MLN cells from *Salmonella*-infected WT or ΔILC2 mice ex vivo, we found that cells from ΔILC2 mice expressed higher levels of transcripts for a diverse array of cytokine outputs (Fig. 5c). These outputs were not limited to any single canonical class, suggesting global dysregulation of cytokine production. Because *Cish* transcripts were not decreased among bulk MLN cells in ΔILC2 mice compared to WT, observed changes in cytokine production were unlikely to be explained by off-target deletion of CISH in non-ILC2s or by enrichment of ILC2s in the MLN cultures (Fig. 5c). When we further examined ΔILC2 or WT mice, we found that the ceca of ΔILC2 mice were grossly shrunken and contracted, with crypt lengthening and prominent lamina propria inflammatory infiltrates evident on histopathology (Fig. 5d).

ILC2s may impact the dissemination of and immune response to *Salmonella* by altering the polarization, differentiation, or behavior of other cell types in the intestine, such as hematopoietic or epithelial cells, including tuft cells. However, we observed no differences in *Salmonella* burden in the MLN of tuft cell-deficient Pou2f3^−/−^ mice compared to WT mice (Fig. 5e). Because Pou2f3^−/−^ mice have reduced tonic intestinal ILC2 activation as a consequence of the loss of tuft cells, we dissociated the role of tuft cells from that of ILC2s by treating Pou2f3^−/−^ or WT mice with IL-25 to activate intestinal ILC2s and mimic the ILC2 hyperactivation observed in ΔILC2 mice. This treatment recapitulated the increased MLN bacterial burden imparted by CISH deletion in ILC2s, but as expected, was not dependent on the presence of tuft cells (Fig. 5e). When we quantified intracellular bacterial burdens from fractionated intestinal cells of *Salmonella*-infected mice, we found that the majority of live, intracellular bacteria were associated with the lamina propria (LP) fraction—specifically from CD45+ cells in the LP—rather than from the epithelial fraction (Fig. 5f). Together, these data suggested that the heightened bacterial burden in ΔILC2 mice was likely attributable to alterations in CD45+ lamina propria populations rather than to the direct infection of epithelial populations.

*S. typhimurium* is a facultative intracellular organism that can disseminate or establish persistent infection by infecting macrophages and dendritic cells.^40^ ILC2s can promote the alternative activation of macrophages:^41,42^ a state in which those macrophages are impaired in their ability to kill intracellular bacteria, thereby increasing the risk of bacterial dissemination and/or persistence.^40,43^ Indeed, sorted LP macrophages from uninfected (naïve) ΔILC2 mice showed significantly increased expression of *Chil3* and *Retnla* and a trend towards increased expression of *Clec10a* and *CD36*, genes associated with “alternative” or “M2” polarization, when compared to macrophages from WT mice (Fig. 5g), while expression of “M1” markers such as *Nos2*, *Ccl2*, *Cxcl9* and *Cxcl10* did not differ (Fig. 5g). Consistent with the previously described impairment in *Salmonella* killing by M2-polarized macrophages, LP macrophages derived from naive ΔILC2 mice also retained more live, intracellular bacteria after in vitro infection than did those from WT mice (Fig. 5h), indicating an intrinsic defect in LP macrophages in ΔILC2 mice. M2 macrophage polarization by ILC2s is promoted through the secretion of IL-13, which acts through a heterodimeric receptor made up of IL4Rα and IL13Rα1.^44^ Therefore, we hypothesized that mice lacking IL-13 signaling through IL4Rα would have decreased M2 polarization among resting LP macrophages, and be better suited to kill and contain *Salmonella* at the mucosal barrier. Indeed, *Salmonella*-infected Il4ra^−/−^ mice had reduced bacterial burden in the MLN and spleen (Fig. 5i, j). Cumulatively, these data are consistent with a model in which CISH constrains ILC2 activity in the intestine, reduces tonic type 2 tone at this critical barrier, and modulates the resistance to potential mucosal pathogenic challenges.

To examine further the role of CISH in modulating ILC2 activity in diverse inflammatory settings, we challenged WT or *Cish*-deficient mice using a model of severe influenza pneumonia. ΔILC2 and KO mice exhibited improved survival after infection when compared to WT mice (Supplementary Fig. 6a). Clinical examination of ΔILC2 and WT mice after infection revealed that ΔILC2 mice lost less body weight, exhibited less hypothermia, and had less severe oxygen desaturation during the peak of infection (Supplementary Fig. 6b–d). As expected, ΔILC2 mice showed evidence of increased ILC2 activity as reflected by IL-13 expression by lung ILC2s and by IL-5 expression in whole lung tissue at the peak of infection (day 7), as well as by ILC2 expansion during the resolution phase (day 14), as compared to WT mice (Supplementary Fig. 6e–i). This data is broadly consistent with prior data suggesting that IL-33-activated ILC2s may promote tissue repair^45^ and blunt influenza-induced immunopathology.

## Discussion

Here, we report that CISH is highly and constitutively expressed in tissue ILC2s and constrains their proliferation, activation and cytokine production. Rather than accompanying activation, CISH expression in ILC2s appears associated with maturation in tissues, as it is increased during development from lymphoid progenitors in bone marrow^46^ and further augmented in peripheral tissue compared to the bone marrow, and coincident with the initiation of effector outputs such as IL-13 and IL-5. Notably, CISH does not prevent these cells from maintaining their poised state. Deficiency of CISH in ILC2s leads to augmented numbers of intestinal tuft cells and fosters heightened epithelial immune tone that facilitates immunity during challenge with intestinal helminths. The increased type 2 immune tone conferred by deficiency of CISH in ILC2s has tradeoffs, however, impacting both antimicrobial resistance and disease tolerance to pathogen challenges that require dominant type 1 or type 17 outputs.

All of the mice used in these experiments were colonized with *Tritrichomonas muris* (*Tm*), an anaerobic, cecal-dwelling protist symbiont that colonizes many mouse SPF research colonies.^47^ *Tm* activates the tuft cell – ILC2 circuit through succinate, a metabolic end-product which binds the G protein-coupled receptor, Sucnr1, on tuft cells, causing them to release IL-25 and activate lamina propria ILC2s, which constituitively express the IL-25 receptor, IL17RB.^30,48^ In this context, tuft and goblet cells were increased in CISH-deficient as compared to WT mice, revealing a role for CISH in restricting IL-25-dependent ILC2 activation. We further confirmed enhanced activation of this pathway by administration of exogenous IL-25 in vivo. Whereas basal survival cytokines like IL-2 and IL-7 are known to activate STAT5 and are therefore anticipated to be enhanced by the loss of feedback inhibition from CISH, IL-25 itself might also activate STAT5^49^ and be similarly subject to CISH-mediated inhibition. Such modulation of IL-25 signaling in the setting of high constitutive expression of IL17RB on intestinal as compared to other tissue ILC2s^20^ would be consistent with the prominent effects of CISH deficiency seen in the small intestine in situ. As such, CISH may play a particularly important role in IL17RB-expressing ILC2s—which include not only those in small intestine, but potentially bone marrow and inflammatory ILC2s that are driven into the blood in response to tissue perturbation.^20,26,31^ Of note, observed changes in ILC2 function in the absence of CISH are unlikely to be entirely dependent on *Tm*-induced IL-25 secretion, since CISH-deficient lung ILC2s cultured in vitro had augmented proliferation and cytokine production in the absence of such stimulation.

In addition to the role we describe for CISH in ILC2s, our data suggests a role for CISH in controlling other diverse cell types, consistent with prior reports.^14,15,17,18^ Specifically, ILC2-intrinsic loss of CISH (ΔILC2) was sufficient to augment ILC2 cytokine production and increase the number of intestinal tuft cells, but neither conditional deficiency of CISH in ILC2s or T cells could recapitulate the early pulmonary hypereosinophilia or degree of reduced worm burden seen in *N.b*.-infected KO mice. Prior reports have identified complementary roles for tissue resident and circulating immune cells in recruitment and activation of pulmonary eosinophilia, as well as in epithelial remodeling,^50^ and CISH activity in T cells and ILC2s could coordinately control these roles. Additionally, CISH is known to restrict eosinophil activity, both by modulating production of eosinophil chemoattractants by stromal cells^51^ and through actions in eosinophils themselves.^52^ Thus, CISH likely functions broadly across diverse cell types to limit coordinated type 2 immune responses during *N.b*. infection.

Many existing data have demonstrated that CISH acts across cell types including NK cells, T cells, dendritic cells, and others^14,15,17,18^ to broadly suppress inflammatory outputs. However, a remaining unresolved issue is why animals that lack CISH globally across all of these cell types demonstrate a type 2 bias.^18^ Humans with single nucleotide polymorphisms conferring reduced function of CISH show increased susceptibility to hepatitis B virus (HBV), bacteremia, tuberculosis (TB), and malaria^53–55^—observations consistent with our finding that CISH-deficient mice have increased susceptibility to *Salmonella* infection—but to our knowledge have not been reported to have allergic predilections. One proposed hypothesis for suppressed immunity in humans with *CISH* polymorphisms is over-stimulation of regulatory T cells, though this hypothesis has not been completely explored. Our data showing alterations in model bacterial and viral infections in both mice that are globally deficient in CISH and ΔILC2 mice suggests that augmented tissue tone established by resident ILC2s contributes to impaired type 1 and type 17 immunity, though this also has not been explored in humans. Additionally, the different microorganisms to which humans versus laboratory rodents under SPF conditions are exposed (such as *Tritrichomonas muris*) might substantially impact how CISH shapes global immune tone. Towards that end, an examination of CISH polypmorphisms among humans who live in regions where TB, malaria, and HBV are not endemic would also be informative.

In sum, we demonstrate that CISH is highly and constitutively expressed in mature tissue ILC2s, where it restrains the activity of these potent effector cells during homeostasis and during immune challenge. The alterations imposed by CISH deficiency in ILC2s are particularly evident in the small intestine in laboratory mice, where loss of CISH in ILC2 shifts the epithelial composition and augments defense against intestinal helminths. However, augmented type 2 immunity comes with tradeoffs, as loss of CISH in ILC2 affects immunity and tolerance to diverse pathogen challenges.

## Methods

### Mice

Arg1^YFP^,^56^ IL5^RFP-Cre^ (“R5”),^23^ IL13^Sm13^ (“S13”),^57^ Il4ra^−/−^,^27^ Pou2f3^−/−^,^30^ and Il25^−/−^/Crlf2^−/−^/Il1rl1^−/−^ (“TKO”)^20^ mice on a C57BL/6J background have been described. Cish^tm1a(KOMP)Wtsi^ mice were obtained from the knockout mouse project produced by the Wellcome Trust Sanger Institute.^58^ These were crossed to either a CMV-Cre (Jax 006054) to knockout CISH in all tissues (KO), or Flippase (Jax 009086) to create a conditional knockout (“CISH^fl/fl^”). After Cre and Flippase were bred out of the lines, subsequent crossing to R5 and CD4-Cre^59^ generated conditional knockouts (“ΔILC2” and “ΔCD4”) in IL-5-expressing cells including ILC2s, or in T cells, respectively. As in Fig. 2a, all mice used to compare WT with ΔILC2 or KO were heterozygous for the IL5^RFP-Cre^ allele to allow for identification of ILC2s and control for the loss of IL-5 expression from the knockin/knockout allele. Concurrent homozygous expression of the Arg1^YFP^ allele (which is expressed by all lung ILC2s but few intestinal ILC2s) aided in identification of ILC2s, while heterozygous expression of the IL13^Sm13^ allele allowed for quantification of IL-13 expression. WT, CISH^fl/fl^ and KO mice used for CD4-Cre-driven conditional deletion were all on Arg1^YFP/YFP^ x IL13^Sm13/Sm13^ dual reporter background. Mice were housed under specific pathogen-free conditions in individually ventilated cages with autoclaved bedding on 12 h light/day cycles and *ad libitum* access to irradiated food (PicoLab Mouse Diet 20, 5058M) and autoclaved water. All animals were manipulated using standard procedures including filtered air exchange stations, chlorine-based disinfection of gloves and work surfaces within manipulations with animals; personnel protection equipment (PPE) (disposable gowns, gloves, head caps, and shoe covers) is required to enter the facility. Experiments were performed on age- and sex-matched male and female mice between 6-12 weeks of age.

### Mouse infections and treatments

Infectious third-stage *Nippostrongylus brasiliensis* (*N.b*.) larvae (L3) were maintained as described.^57^ Mice were infected subcutaneously with 500 *N.b*. L3 and sacrificed on day 5 or 11 of infection to collect tissues for staining or to count intestinal worm burden, as described.^57^ To assess lung injury attributable to migratory worms, bronchoalveolar lavage (BAL) was performed by intratracheal instillation and recovery of 1.5 mL of saline at day 3 of infection. IL-25-treated mice were injected intraperitoneally with 200 ng of carrier-free recombinant mouse IL-25 (BioLegend) on days 0, 1, and 2, and sacrificed on day 3 for analysis. For *Salmonella* infection followed by quantification of CFU from fractionated small intestinal cells, mice were fasted for 4 h, pretreated with streptomycin by oral gavage 24 h prior to inoculation with 10^7^ CFU of *Salmonella typhimurium* L1344 by oral gavage, and then sacrified on day 2. In comparison to the standard infection model, this dose and timing was chosen to maximize both cell viability during the sort, and bacterial recovery. For all other *Salmonella* infections, mice were fasted for 4 h, pretreated with streptomycin by oral gavage 24 h prior to inoculation with 10^6^ CFU of *Salmonella typhimurium* L1344 by oral gavage, and then sacrified on day 3. Titered PR8 influenza was generously provided by Dr. Jeff Gotts at UCSF. Mice were infected intranasally under isofluorane anesthesia with 800 focus forming units (FFU) of influenza virus. Body weight, rectal temperature, and oxygen saturation were measured using the MouseOx oximeter and software (Starr Life Sciences) at the indicated timepoints.

### Cell isolation & flow cytometry

After removal of Peyer’s patches, single cell suspensions from small intestine were prepared by serial washing in neutral buffer containing EDTA and DTT to remove epithelial cells, followed by digestion with 0.1 Wunsch/mL LiberaseTM (Roche) and 30 μg/mL DNAse I (Roche) to isolate lamina propria cells. Single cell suspensions from the skin were made by incubating equal-sized patches of minced, shaved skin from the animals’s back in 1.25 mg/mL LiberaseTL (Roche) and 100 μg/mL DNAse I. Lung single cell suspensions were obtained by tissue digestion with 50 μg/mL LiberaseTM and 25 μg/mL DNAse I. All suspensions were depleted of red blood cells using PharmLyse (BD) and passed through a 40 μM strainer prior to staining. ILC2s were gated as lin–, Thy1.2+, Arg1^YFP^+, R5+. A representative gating strategy for ILC2s is shown in Supplementary Fig. 2a. Tuft cells were defined as DCLK1+, CD44–, EpCAM+, CD45–. Lineage cocktail and other antibodies used for flow cytometry are listed in Supplementary Table 1. After antibody staining, flow cytometry was performed on a LSR Fortessa (BD Biosciences) using BD FACSDiva software and analyzed using FlowJo software (Tree Star). Dead cells were excluded from analysis based on staining with LIVE/DEAD Viability (Thermo) or 4′,6-diamidino-2-phenylindole (DAPI).

For CFU from fractionated intestinal cells as in Fig. 5f, the cells isolated during the EDTA/DTT wash and the cells isolated after the Liberase/DNAse digestion (as described in the preceding paragraph) were separately stained, cleared of extracellular bacteria by treatment with ampicillin, and then sorted on a MoFlo Cell Sorter (Beckman Coulter) according to CD45 expression. Lamina propria macrophages (used for expression analysis or in vitro infection) were defined as CD45+, CD64+, CD11b+.

### Cell culture

ILC2s were isolated from lung single cell suspensions on a MoFlo Cell Sorter (Beckman Coulter). For in vitro stimulation in Fig. 1b, lung ILC2s were gated as CD45+, lineage (“lin”; CD4, CD8, CD3, CD11b, CD11c, CD19, NK1.1, NKp46, Gr-1, F4/80, Ter119, DX5) negative, Thy1.2+, Sca1+, Arg1^YFP^+ and intestinal ILC2s were gated as CD45+, lin–, KLRG1+, Sca1+. For all other experiments, ILC2s were gated as lin–, Thy1.2+, Arg1^YFP^+, R5+. This sorting method routinely yields a purity of ≥88–98% (from a pre-sort frequency of ~0.5%). Purified cells were cultured at a density of 5 × 10^3^ (for short-term culture) or 9 × 10^3^ (for multi-day culture) per well in 200 μL of RPMI media supplemented with 10% heat-inactivated FBS, 10 mM HEPES (Sigma), pH 7.4, penicillin–streptomycin (Gibco), 2-mercaptoethanol (Gibco), and IL-2 and IL-7 at 10 ng/mL each (R&D Systems). Stimulations were performed with addition of 10 ng/mL IL-25 (BioLegend), 10 ng/mL IL-33 (BioLegend), or 40 ng/mL phorbol 12-myristate 13-acetate (PMA) (Cayman) and 500 ng/mL ionomycin (Sigma) for 4 h at 37 °C. For MLN cultures, equal numbers of dissociated cells were restimulated in supplemented RPMI containing IL-2 at 10 ng/mL, PMA/ionomycin, and ampicillin overnight before lysis in RLT-plus buffer (Qiagen). For proliferation studies, cells were stained with 100 nM CellTrace Far Red dye (Thermo) for 10 min prior to cultures. Supernatants were collected after centrifugation for protein analysis, and cell pellets were either lysed in RLT-plus for later RNA isolation, or resuspended and stained for flow cytometric analysis. IL-5 and IL-13 protein were quantified in supernatants using Cytometric Bead Array Flex Sets, acquired with a LSR Fortessa, and analyzed using Flow Cytometric Analysis Program (FCAP) Array software (BD Biosciences).

For in vitro macrophage infections, 2.5 × 10^5^ lamina propria macrophages (as described in “cell isolation & flow cytometry”) were separately isolated from 3 mice of each genotype and allowed to adhere to 24 well plates for 2 h prior to inoculation with *S. typhimurium* at MOI = 100 in RPMI media supplemented with 10% heat-inactivated FBS, 10 mM HEPES (Sigma), pH 7.4, penicillin–streptomycin (Gibco), 2-mercaptoethanol (Gibco). Extracellular bacteria were killed by addition of gentamicin 1 h after infection, after which macrophages were cultured overnight, then lifted from TC plates, washed twice to remove any remaining extracellular bacteria, and plated for CFU quantification.

### Histology & immunofluorescence

The most proximal 2 cm of small intestine (SI) were discarded and the next 6 cm were cleared of luminal content, and fixed in 4% paraformaldehyde for 4 h at 4 °C followed by PBS wash. Tissues were cryoprotected overnight in 30% (w/v) sucrose before embedding in OCT media (Sakura). 8 μm frozen sections were prepared on a cryostat (Leica), stained with antibodies as listed in Supplementary Table 1, counterstained with DAPI, mounted, and analyzed on a Nikon A1R confocal microscope using NIS Elements (Nikon) and Fiji (ImageJ) software. For histologic sections of small intestine or cecum, SI samples (harvested and fixed as above) or whole PFA-fixed cecum were embedded, sectioned, and stained with hematoxylin and eosin (H&E) or Alcian blue - Periodic Acid-Schiff (PAB) at the UCSF Histology and Biomarker Core, and imaged on a Nikon A1R microscope.

### RNA expression analysis

Comparative expression of *Cish* in multiple lymphoid subsets was investigated through the publicly available browser created and sourced by the Immunological Genome Project (ImmGen).^19^ Gene expression of *Cish* and related transcripts in ILC2s or comparator lymphocyte populations from various tissues of wild type mice was performed as described,^20^ and previously made available at Gene Expression Omnibus (GEO) under accession code GSE117568.

For RT-PCR, ILC2s or CD4+ T cells from lung (~10,000–20,000 cells per mouse) of 3 mice per group were sorted individually into RLT Plus lysis buffer (Qiagen) and stored at –80 °C, then processed using the RNeasy Micro Plus kit (Qiagen) per the manufacturer’s protocol. RNA was reverse transcribed using the SuperScript VILO cDNA synthesis kit (ThermoFisher) and amplified using Power SYBR Green PCR master mix (ThermoFisher) using the primers listed in Supplementary Table 2. Expression was quantified using the delta-delta Ct method normalized to housekeeping genes (*Rpl13a* and *Hprt*).

For RNA sequencing experiments, lung and SI ILC2s were purified from tissue as described above and collected directly into lysis buffer provided in the DynaBead Direct mRNA Micro Kit (ThermoFisher). Poly-adenylated mRNA was purified according to the manufacturer’s instructions, and sequenced by Tag-Seq method on HiSeq 4000 (Illumina) at the UC Davis Genomics Core. For whole tissue RNA sequencing, 1 cm of intact small intestine, cleared of mesentery and luminal content, was homogenized in RNAzol (Sigma), isolated by 4-bromoanisole phase separation according to the manufacturer’s instructions, and then cleaned up using an RNeasy Plus Mini Kit (Qiagen) before submission for Tag-Seq at the UC Davis Genomics Core. Reads were aligned to the mouse genome and quantified using the STAR aligner software version 2.7.2b. Differential expression analysis was performed in the R computing environment version 3.6.1 using the software DESeq2 version 1.26. Significance thresholds used were FDR < 0.05 and log fold change >1 or <−1. Pathway analysis was performed using the Gene Set Enrichment Analysis software (Broad Institute).^60,61^ Sequencing datasets will be made publically available through the NCBI Gene Expression Ombibus (GEO).

### Statistical analysis

All graphical representations and statistical analyses were performed with Graphpad Prism 8 or 9 using the appropriate statistical tests with posthoc testing as specified in figure legends. Figures display means ± SEM as indicated.

### Study approval

All experimental procedures on mice were approved by the UCSF Animal Care and Use Committee.

## Supplementary information

Supplementary Table 1

Supplementary Material

Supplementary Table 3

Supplementary Table 4

## References

[1] Klose CS, Artis D (2016). Innate lymphoid cells as regulators of immunity, inflammation and tissue homeostasis. Nat. Immunol..

[2] Kotas ME, Locksley RM (2018). Why innate lymphoid cells?. Immunity.

[3] Sivori S (2019). Human NK cells: surface receptors, inhibitory checkpoints, and translational applications. Cell Mol. Immunol..

[4] Cerwenka A, Lanier LL (2016). Natural killer cell memory in infection, inflammation and cancer. Nat. Rev. Immunol..

[5] Duerr CU (2016). Type I interferon restricts type 2 immunopathology through the regulation of group 2 innate lymphoid cells. Nat. Immunol..

[6] Moro K (2016). Interferon and IL-27 antagonize the function of group 2 innate lymphoid cells and type 2 innate immune responses. Nat. Immunol..

[7] Nagashima H (2019). Neuropeptide CGRP limits group 2 innate lymphoid cell responses and constrains type 2 inflammation. Immunity.

[8] Moriyama S (2018). Beta2-adrenergic receptor-mediated negative regulation of group 2 innate lymphoid cell responses. Science.

[9] Garg AV, Ahmed M, Vallejo AN, Ma A, Gaffen SL (2013). The deubiquitinase A20 mediates feedback inhibition of interleukin-17 receptor signaling. Sci. Signal.

[10] Trengove MC, Ward AC (2013). SOCS proteins in development and disease. Am. J. Clin. Exp. Immunol..

[11] Yoshimura A (1995). A novel cytokine-inducible gene cis encodes an Sh2-containing protein that binds to tyrosine-phosphorylated interleukin-3 and erythropoietin receptors. Embo J..

[12] Matsumoto A (1999). Suppression of STAT5 functions in liver, mammary glands, and T cells in cytokine-inducible SH2-containing protein 1 transgenic mice. Mol. Cell Biol..

[13] Matsumoto A (1997). CIS, a cytokine inducible SH2 protein, is a target of the JAK-STAT5 pathway and modulates STAT5 activation. Blood.

[14] Palmer DC (2015). Cish actively silences TCR signaling in CD8+ T cells to maintain tumor tolerance. J. Exp. Med.

[15] Delconte RB (2016). CIS is a potent checkpoint in NK cell-mediated tumor immunity. Nat. Immunol..

[16] Zhu H (2020). Metabolic reprograming via deletion of CISH in human iPSC-derived NK cells promotes in vivo persistence and enhances anti-tumor activity. Cell Stem Cell.

[17] Miah MA (2012). CISH is induced during DC development and regulates DC-mediated CTL activation. Eur. J. Immunol..

[18] Yang XO (2013). The signaling suppressor CIS controls proallergic T cell development and allergic airway inflammation. Nat. Immunol..

[19] Heng TS, Painter MW (2008). Immunological Genome Project C. The Immunological Genome Project: networks of gene expression in immune cells. Nat. Immunol..

[20] Ricardo-Gonzalez RR (2018). Tissue signals imprint ILC2 identity with anticipatory function. Nat. Immunol..

[21] Price AE (2010). Systemically dispersed innate IL-13-expressing cells in type 2 immunity. Proc. Natl Acad. Sci. USA.

[22] Molofsky AB (2013). Innate lymphoid type 2 cells sustain visceral adipose tissue eosinophils and alternatively activated macrophages. J. Exp. Med.

[23] Nussbaum JC (2013). Type 2 innate lymphoid cells control eosinophil homeostasis. Nature.

[24] Halim TY, Krauss RH, Sun AC, Takei F (2012). Lung natural helper cells are a critical source of Th2 cell-type cytokines in protease allergen-induced airway inflammation. Immunity.

[25] Ricardo-Gonzalez, R. R. et al. Tissue-specific pathways extrude activated ILC2s to disseminate type 2 immunity. *J. Exp. Med.* **217**, e20191172 (2020). 10.1084/jem.20191172 PMC7144525 32031571

[26] Huang Y (2015). IL-25-responsive, lineage-negative KLRG1(hi) cells are multipotential ‘inflammatory’ type 2 innate lymphoid cells. Nat. Immunol..

[27] von Moltke J, Ji M, Liang HE, Locksley RM (2016). Tuft-cell-derived IL-25 regulates an intestinal ILC2-epithelial response circuit. Nature.

[28] Gerbe F (2016). Intestinal epithelial tuft cells initiate type 2 mucosal immunity to helminth parasites. Nature.

[29] Howitt MR (2016). Tuft cells, taste-chemosensory cells, orchestrate parasite type 2 immunity in the gut. Science.

[30] Schneider C (2018). A metabolite-triggered tuft cell-ILC2 circuit drives small intestinal remodeling. Cell.

[31] Huang Y (2018). S1P-dependent interorgan trafficking of group 2 innate lymphoid cells supports host defense. Science.

[32] Haber AL (2017). A single-cell survey of the small intestinal epithelium. Nature.

[33] Chen HW, Heiniger HJ, Kandutsch AA (1975). Relationship between sterol synthesis and DNA synthesis in phytohemagglutinin-stimulated mouse lymphocytes. Proc. Natl Acad. Sci. USA.

[34] Russell DW (1992). Cholesterol biosynthesis and metabolism. Cardiovasc Drugs Ther..

[35] Bietz A, Zhu H, Xue M, Xu C (2017). Cholesterol metabolism in T cells. Front. Immunol..

[36] MacIver NJ, Michalek RD, Rathmell JC (2013). Metabolic regulation of T lymphocytes. Annu. Rev. Immunol..

[37] Kidani Y (2013). Sterol regulatory element-binding proteins are essential for the metabolic programming of effector T cells and adaptive immunity. Nat. Immunol..

[38] Desai P (2021). Enteric helminth coinfection enhances host susceptibility to neurotropic flaviviruses via a tuft cell-IL-4 receptor signaling axis. Cell.

[39] Broz P, Ohlson MB, Monack DM (2012). Innate immune response to Salmonella typhimurium, a model enteric pathogen. Gut Microbes.

[40] Eisele NA (2013). Salmonella require the fatty acid regulator PPARdelta for the establishment of a metabolic environment essential for long-term persistence. Cell Host Microbe.

[41] Bouchery T (2015). ILC2s and T cells cooperate to ensure maintenance of M2 macrophages for lung immunity against hookworms. Nat. Commun..

[42] Besnard AG (2015). IL-33-mediated protection against experimental cerebral malaria is linked to induction of type 2 innate lymphoid cells, M2 macrophages and regulatory T cells. PLoS Pathog..

[43] Pham THM (2020). Salmonella-driven polarization of granuloma macrophages antagonizes TNF-mediated pathogen restriction during persistent infection. Cell Host Microbe.

[44] Gordon S, Martinez FO (2010). Alternative activation of macrophages: mechanism and functions. Immunity.

[45] Monticelli LA (2011). Innate lymphoid cells promote lung-tissue homeostasis after infection with influenza virus. Nat. Immunol..

[46] Seillet C (2016). Deciphering the innate lymphoid cell transcriptional program. Cell Rep..

[47] Chudnovskiy A (2016). Host-protozoan interactions protect from mucosal infections through activation of the inflammasome. Cell.

[48] Nadjsombati MS (2018). Detection of succinate by intestinal tuft cells triggers a type 2 innate immune circuit. Immunity.

[49] Wu L (2015). A novel IL-25 signaling pathway through STAT5. J. Immunol..

[50] Rahimi, R. A., Nepal, K., Cetinbas, M., Sadreyev, R. I., Luster, A. D. Distinct functions of tissue-resident and circulating memory Th2 cells in allergic airway disease. *J. Exp. Med.* **217**, e20190865 (2020). 10.1084/jem.20190865 PMC7478729 32579670

[51] Takeshima H (2019). CISH is a negative regulator of IL-13-induced CCL26 production in lung fibroblasts. Allergol. Int..

[52] Burnham ME (2013). Human airway eosinophils exhibit preferential reduction in STAT signaling capacity and increased CISH expression. J. Immunol..

[53] Khor CC (2010). CISH and susceptibility to infectious diseases. N. Engl. J. Med..

[54] Tong HV (2011). Association of CISH -292A/T genetic variant with hepatitis B virus infection. Immunogenetics.

[55] Sun L (2014). Genetic contribution of CISH promoter polymorphisms to susceptibility to tuberculosis in Chinese children. PLoS One.

[56] Reese TA (2007). Chitin induces accumulation in tissue of innate immune cells associated with allergy. Nature.

[57] Liang HE (2011). Divergent expression patterns of IL-4 and IL-13 define unique functions in allergic immunity. Nat. Immunol..

[58] Skarnes WC (2011). A conditional knockout resource for the genome-wide study of mouse gene function. Nature.

[59] Lee PP (2001). A critical role for Dnmt1 and DNA methylation in T cell development, function, and survival. Immunity.

[60] Subramanian A, Kuehn H, Gould J, Tamayo P, Mesirov JP (2007). GSEA-P: a desktop application for Gene Set Enrichment Analysis. Bioinformatics.

[61] Subramanian A (2005). Gene set enrichment analysis: a knowledge-based approach for interpreting genome-wide expression profiles. Proc. Natl Acad. Sci. USA.

