# Supplementary material for: CISH constrains the tuft–ILC2 circuit to set epithelial and immune tone
Source: Mucosal Immunol. 2021 Jul 21;14(6):1295–305. doi: 10.1038/s41385-021-00430-6 (PMC8528700; doi:10.1038/s41385-021-00430-6)
Supplement: Supplementary file 2 — Supplementary Material [file 41385_2021_430_MOESM2_ESM.pdf]

Supplementary Material

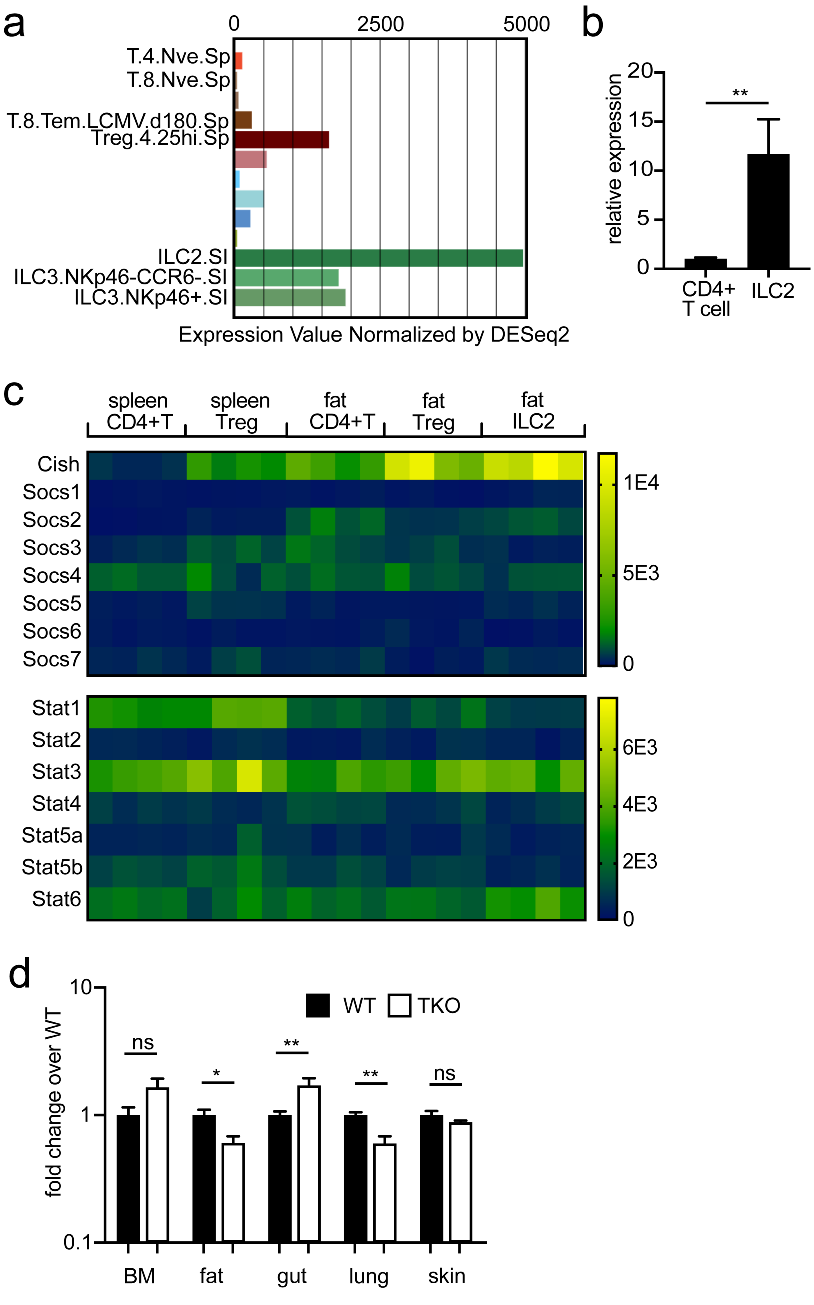

Supplementary Figure 1: CISH is highly expressed in tissue ILC2s. **a** *Cish* is highly expressed in ILC2s compared to other leukocytes. Data adapted from ImmGen. **b** Expression of *Cish* in lung ILC2s or CD4+ T cells. **p<0.01 by 2-tailed t-test. n = 4 mice/tissue. **c** RNA sequencing from purified ILC2s from multiple tissues ILC2s as compared to other tissue resident lymphocytes.

Legend colors indicate counts per million reads. **d** *Cish* expression in WT or $Il25^{-/-}/Crlf2^{-/-}/Il1rl1^{-/-}$ ("TKO") mice. *p<0.05, **p<0.01, ns=non-significant by unpaired t-test. n = 3-7 mice/group.

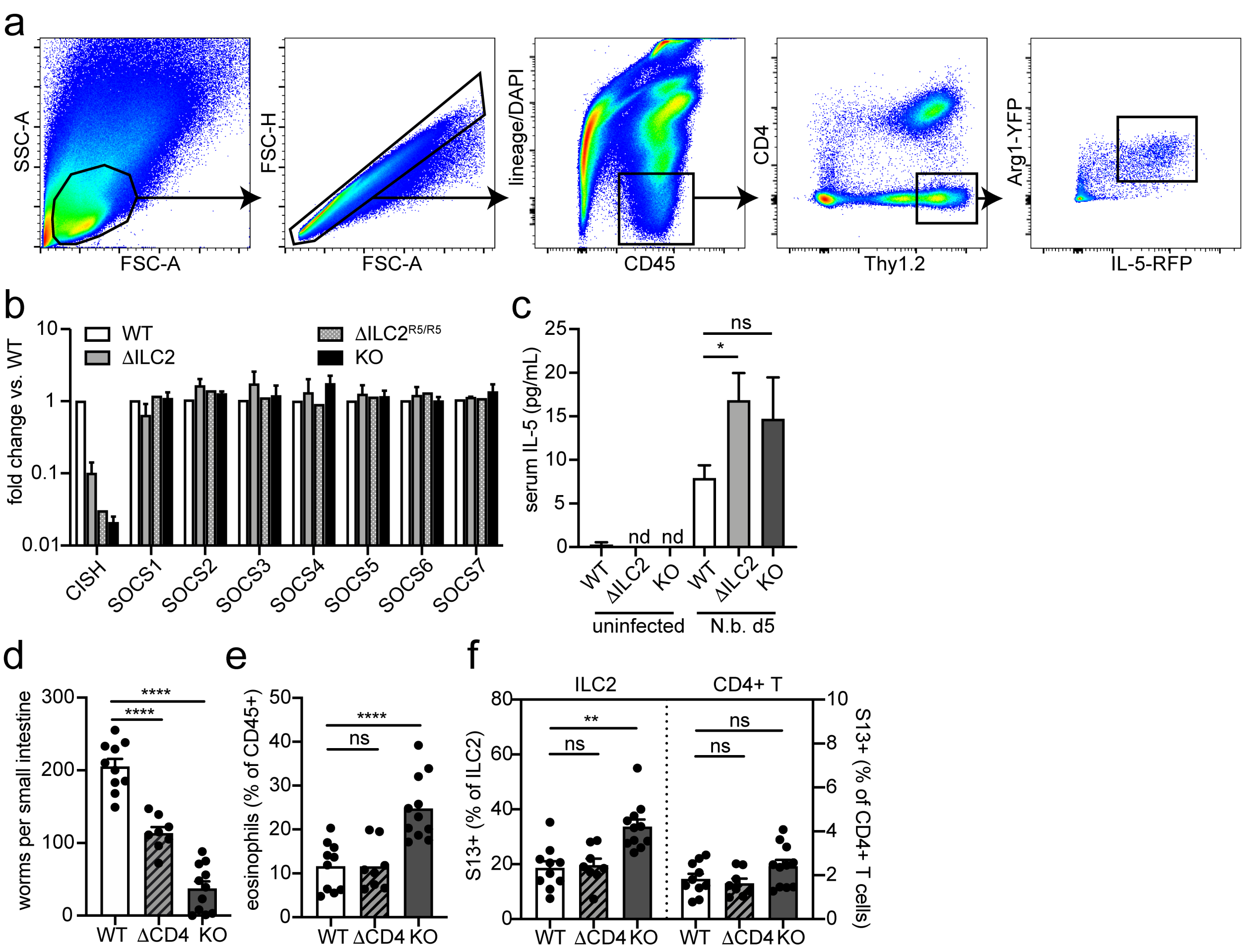

<u>Supplementary Figure 2: CISH knockdown in ILC2s or T cells leads to augmented immunity to helminth challenge.</u> **a** Representative gating strategy for lung ILC2s. **b** Expression of *Cish* and other SOCS family members in lung ILC2s measured by qPCR. $\Delta ILC2^{R5/R5}$ indicates homozygous expression of R5. Statistics shown for *Cish* in Figure 1a; all others non-significant. n = 3 mice/group. **c** Serum IL-5 measured at day 5 of infection with *N.b.* as in Figure 2. *p<0.05. n = 6 mice/group. **d** Number of *N.b.* worms in SI on day 5. ****p<0.0001 for one-way ANOVA with Dunnett testing for multiple comparisons. n = 8-11 mice/group; data pooled from 2 similar experiments. **e** Lung eosinophils on infection day 5. ***p<0.001; ns = non-significant for one-way ANOVA with Dunnett testing for multiple comparisons. n = 8-11 mice/group; data pooled from 2 similar experiments. **f** IL-13 expression (S13) on ILC2s or CD4+ T cells at day 5 of infection.

**p<0.01 for Brown-Forsythe and Welch ANOVA with Dunnett correction for multiple comparison. ns=non-significant. n = 8-11 mice/group pooled from 2 similar experiments.

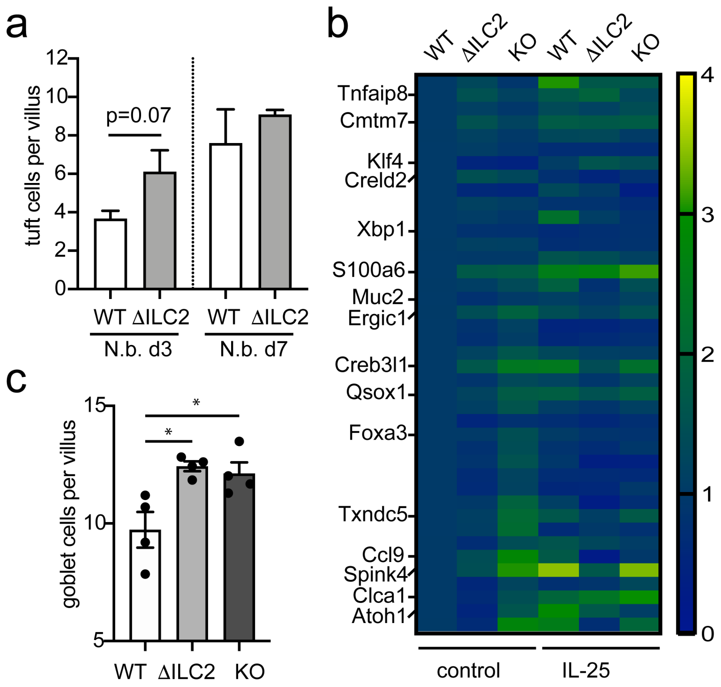

<u>Supplementary Figure 3: CISH constraint of ILC2 outputs controls secretory cell development in the intestine.</u> **a** Tuft cells per villus counted from immunofluorescence staining of intestines from *N.b.*-infected mice at indicated timepoints. Numbers indicate average +/- SEM over a minimum of 10 intact villi from each of 4-5 mice/group. **b** Goblet cell markers in whole intestinal tissue harvested from IL-25- or untreated mice. Each block represents row-normalized mean expression in 3 mice/group. **c** Goblet cells counted from PAB-stained sections of untreated mice of indicated genotypes. Numbers indicate average +/- SEM over a minimum of 10 intact villi from each of 4 mice/group. *P<0.05 by one-way ANOVA with Dunnett testing for multiple comparisons.

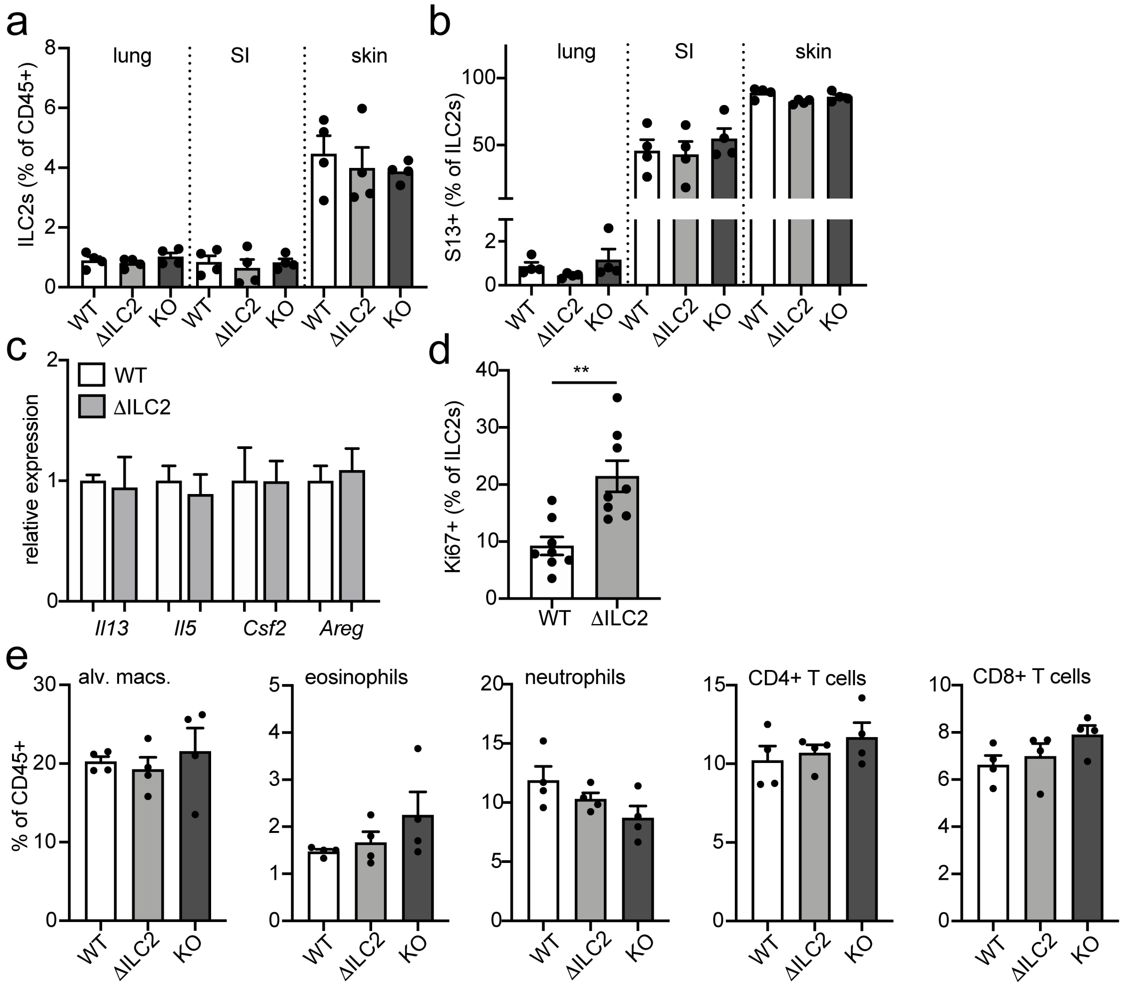

Supplementary Figure 4: Loss of CISH in ILC2s yields increased tissue ILC2 turnover without affecting steady state numbers. **a** Proportion of ILC2s in the lung, SI, and skin. n = 4 mice/group, representative of 2 similar experiments. **b** IL-13 expression in ILC2s in the lung, SI, and skin. n = 4 mice/group. **c** Expression of indicated effector genes in sorted lung ILC2s. n = 3-9 mice/group, pooled from 2 of 3 similar experiments. **d** Ki67 expression measured by flow cytometry from freshly-isolated SI ILC2s. n = 8 mice/group, pooled from 2 similar experiments. **$p<0.01$. **e** Leukocyte subsets (as indicated) in from lung of untreated mice. n = 4 mice/group, representative of 2 similar experiments.

a

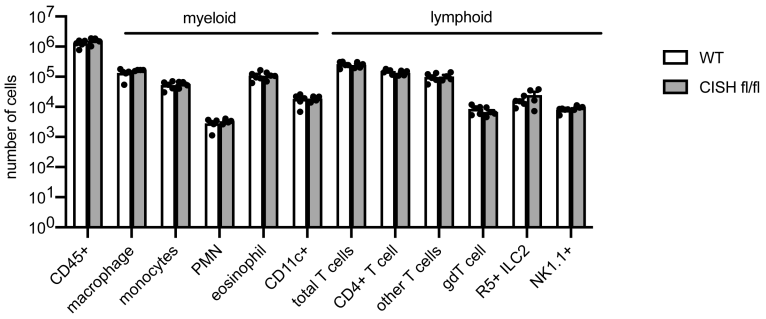

Supplementary Figure 5: Loss of CISH in ILC2s yields no significant differences in numbers of intestinal immune cells during *Salmonella* infection. **a** number of cells counted per centimeter of jejunum.

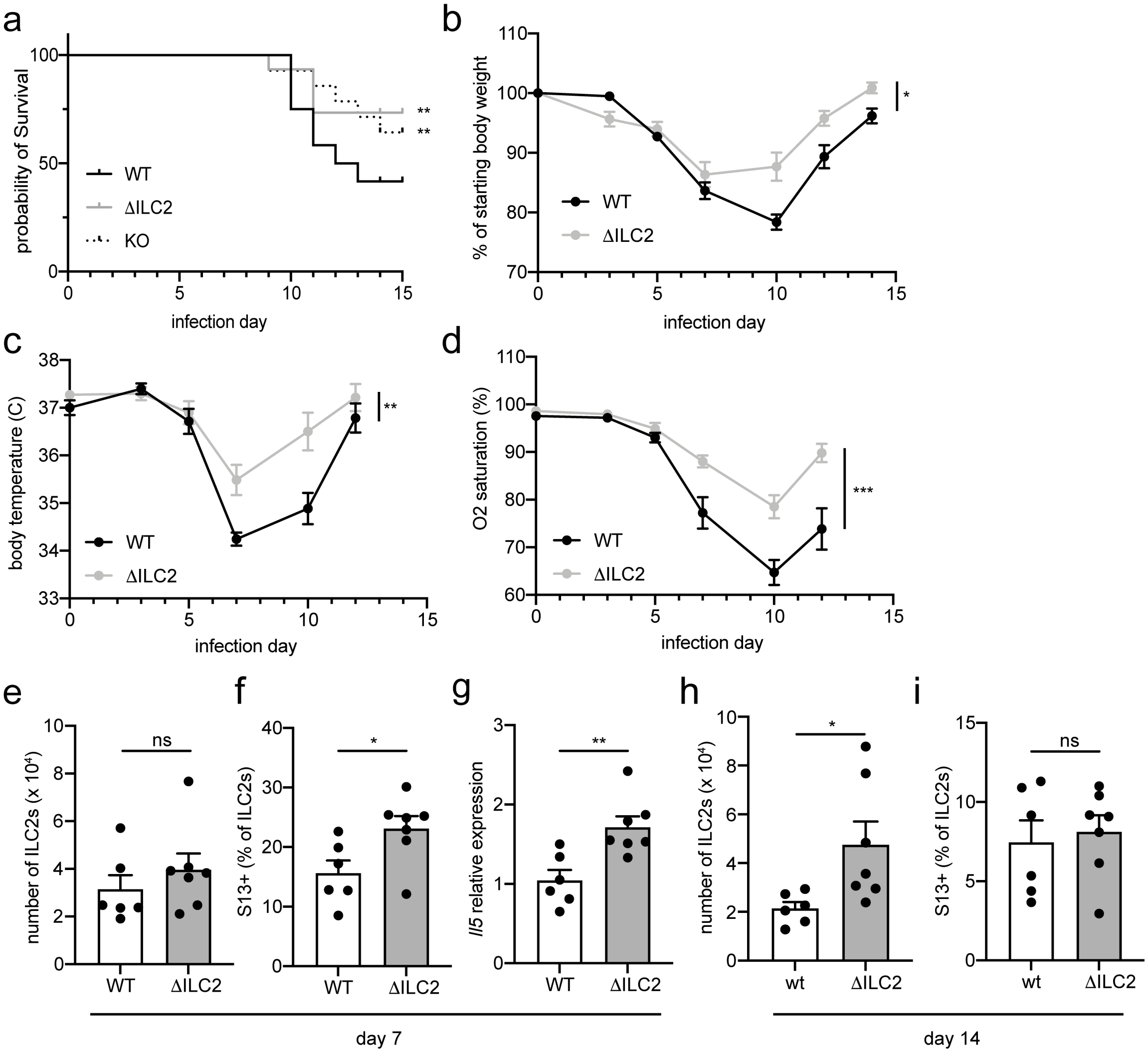

<u>Supplementary Figure 6: Augmented ILC2 activity through CISH deletion improves survival during influenza infection.</u> **a** Survival after influenza PR8 in indicated strains. n = 12-15 mice per group, pooled from two experiments. **b** Body weight, **c** body temperature and, **d** oxygen saturation measured in indicated genotypes. for (a-d), *p<0.05, **p<0.01, ***p<0.001 for differences between genotype by 2-way ANOVA. n = 7 mice/group. **e** Number of lung ILC2s and **f** S13 reporter expression on lung ILC2s on day 7 of influenza infection. **g** *Il5* transcripts in whole lung tissue on day 7 of influenza infection. **h** number of lung ILC2s and **i** S13 reporter

expression on lung ILC2s on day 14 of influenza infection. for (e-i), *$p<0.05$, **$p<0.01$, ns = non-significant by t-test. n = 6-7 mice/group.

Supplementary Table 1: Antibodies used

Supplementary Table 2: Primers used for qRT-PCR

**Gene symbol**	**Forward Primer**	**Reverse Primer**
Areg	CAGCTATTGGCATCGGCATC	TTCAACTTTTACCCTGCATTGTCC
Ccl2	AGGTCCCTGTCATGCTTCTGG	CTGCTGCTGGTGATCCTCTTG
Cd36	GCTTGCAACTGTCAGCACAT	GCCTTGCTGTAGCCAAGAAC
Chil3	CAGGTCTGGCAATTCTTCTGAA	GTCTTGCTCATGTGTGTAAGTGA
Cish	ATGGTCCTTTGCGTACAGGG	GGAATGCCCCAGTGGGTAAG
Clec10a	CAGAATCGCTTAGCCAATGTGG	TCCCAGTCCGTGTCCGAAC
Csf2	TCAGAGAGAAAGGCTAAGGTCC	CTCTTCATTCAACGTGACAGGC
Cxcl10	CCAAGTGCTGCCGTCATTTTC	GGCTCGCAGGGATGATTTCAA
Cxcl9	TCCTTTTGGGCATCATCTTCC	TTTGTAGTGGATCGTGCCTCG
Hprt	GTTGGATACAGGCCAGACTTTGTTG	GAGGGTAGGCTGGCCTATAGGCT
Ifng	CATTGAAAGCCTAGAAAGTCTGAATAAC	TGGCTCTGCAGGATTTTCATG
Il10	GCTGGACAACATACTGCTAACCG	CCTTGCTCTTATTTTCACAGGGG
Il13	GGATATTGCATGGCCTCTGTAAC	AACAGTTGCTTTGTGTAGCTGA
Il17a	AGCAGCGATCATCCCTCAAAG	GTCTTCATTGCGGTGGAGAGTC
Il2	TGAGCAGGATGGAGAATTACAGG	GTCCAAGTTCATCTTCTAGGCAC
Il22	TCAGACAGGTTCCAGCCCTA	CAGGTCCAGTTCCCCAATCG
Il4	GCTCGTCTGTAGGGCTTCC	GTGCAGCTTATCGATGAATCCAG
Il5	CTCTGTTGACAAGCAATGAGACG	TCTTCAGTATGTCTAGCCCCTG
Il9	ATGTTGGTGACATACATCCTTGC	TGACGGTGGATCATCCTTCAG
Nos2	GAATCTTGGAGCGAGTTGTGG	CAGGAAGTAGGTGAGGGCTTG
Retnla	CCAATCCAGCTAACTATCCCTCC	ACCCAGTAGCAGTCATCCCA
Rpl13a	GAGGTCGGGTGGAAGTACCA	TGCATCTTGGCCTTTTCCTT
Tnfa	TCTGTCTACTGAACTTCGGGGTG	ACTTGGTGGTTTGCTACGACG

Supplementary Table 3: List of gene symbols for consensus “tuft cell” and “goblet cell” identities corresponding to heatmaps in Figure 3c and Supplementary Figure 3b

Supplementary Table 4: Selected outputs from Gene Set Enrichment Analysis
